# Psychological distress of patients suffering from restless legs syndrome: a cross-sectional study

**DOI:** 10.1186/1477-7525-9-73

**Published:** 2011-09-20

**Authors:** Hanna Scholz, Heike Benes, Svenja Happe, Juergen Bengel, Ralf Kohnen, Magdolna Hornyak

**Affiliations:** 1 Interdisciplinary Pain Centre, University Medical Centre, Breisacher Strasse 64, Freiburg 79106, Germany; 2 Somni bene Institute for Medical Research and Sleep Medicine Schwerin Ltd, Goethe Strasse 1, Schwerin 19053, and Neurology Department, University of Rostock, Gehlsheimer Strasse 20, Rostock 18147, Germany; 3 Department of Clinical Neurophysiology, Klinikum Bremen-Ost, Züricher Strasse 40, Bremen 28325, and Department of Clinical Neurophysiology, University of Göttingen, Robert-Koch-Strasse 40, Göttingen 37079, Germany; 4 Institute for Psychology, Rehabilitation Psychology and Psychotherapy, University of Freiburg, Engelberger Strasse 41, Freiburg 79085, Germany; 5 Research Pharmaceutical Services Inc., 520 Virginia Drive, Fort Washington PA 19034, USA, and Department of Psychology, University of Erlangen-Nuremberg, Regensburger Strasse 160, Nuremberg 90478, Germany

**Keywords:** restless legs syndrome, psychological impairment, psychopathology, depression, anxiety, compulsivity, somatisation

## Abstract

**Background:**

Restless legs syndrome (RLS) is a chronic disorder with substantial impact on quality of life similar to that seen in diabetes mellitus or osteoarthritis. Little is known about the psychological characteristics of RLS patients although psychological factors may contribute to unfavourable treatment outcome.

**Methods:**

In an observational cross-sectional design, we evaluated the psychological features of 166 consecutive RLS patients from three outpatient clinics, by means of the Symptom Checklist 90-R (SCL-90-R) questionnaire. Additionally, the Beck Depression Inventory-II (BDI-II) and the International RLS Severity Scale (IRLS) were measured. Both treated and untreated patients were included, all patients sought treatment.

**Results:**

Untreated patients (n = 69) had elevated but normal scores on the SCL-90-R Global Severity Index (GSI; p = 0.002) and on the sub-scales somatisation (p < 0.001), compulsivity (p = 0.003), depression (p = 0.02), and anxiety (p = 0.004) compared with a German representative sample. In the treated group, particularly in those patients who were dissatisfied with their actual treatment (n = 62), psychological distress was higher than in the untreated group with elevated scores for the GSI (p = 0.03) and the sub-scales compulsivity (p = 0.006), depression (p = 0.012), anxiety (p = 0.031), hostility (p = 0.013), phobic anxiety (p = 0.024), and paranoid ideation (p = 0.012). Augmentation, the most serious side effect of dopaminergic, i.e. first-line treatment of RLS, and loss of efficacy were accompanied with the highest psychological distress, as seen particularly in the normative values of the sub-scales compulsivity and anxiety. Generally, higher RLS severity was correlated with higher psychological impairment (p < 0.001).

**Conclusion:**

Severely affected RLS patients show psychological impairment in multiple psychological domains which has to be taken into account in the treatment regimen.

## Background

Restless legs syndrome (RLS) is a common neurological disorder in Western countries with a lifetime prevalence of 7 to 10% [1]. Approximately 1 to 3% of patients require treatment [2]. The disease specific, health-related, and psychosocial quality of life of this population is reduced compared to the general population and is comparable to that of patients with type 2 diabetes mellitus and osteoarthritis [2,3]. The lifetime prevalence of comorbid depression and anxiety disorders is elevated by odds ratios of 2.1 to 5.3 in RLS compared to the community at large [4-6]. Sleep problems, leg dysaesthesias, and the psychological sequelae of the disorder are all particularly implicated in contributing to impaired daily functioning [7,8]. RLS is considered to be a chronic disorder as causative treatments do not exist except of a few secondary forms such as iron deficiency. Dopamine agonists, the first-line treatment in RLS, show efficacy which is, however, moderate [9] and the majority of patients do not experience full remission in drug trials [10,11]. Little is known about the psychopathological state and psychological wellbeing of RLS patients. This issue is, however, of major clinical relevance as psychological factors may contribute to an unfavourable treatment outcome as seen for example in chronic pain conditions [12]. One study investigated personality traits, i.e. stable patterns of behaviour, thoughts, and emotions, by using the NEO-Personality Inventory and found elevated neuroticism scores in RLS (n = 42) compared with non-RLS subjects (n = 982) [13].

In the present study we investigated the psychological impairment of RLS patients in a cross-sectional observational design. To evaluate the psychological profile, we used the Symptom-Checklist-90-Revised version (SCL-90-R [14,15]), a broadly used self-report inventory which captures the main dimensions of the actual psychopathology of a person in nine sub-scales and a Global Severity Index (GSI). Additionally, depressive symptoms were assessed using the Beck Depression Inventory.

## Methods

Data of 166 consecutive German patients was collected over a period of 12 months (October 2006 until October 2007). These patients sought treatment for RLS at the RLS outpatient clinic at the University Medical Centre Freiburg (affiliated to the Sleep Disorders Centre of the Dept. of Psychiatry and Psychotherapy to that time; n = 111), in the Sleep Disorders Centre in Schwerin (Somni bene Institute for Medical Research and Sleep Medicine; n = 15) and the Sleep Disorders Centre of the Department of Clinical Neurophysiology in Bremen (n = 40). A detailed description of the patient population, including comorbidity and medication, is presented in the *Results *section.

Diagnosis was made according to valid diagnostic criteria (IRLSSG [16]) in a face-to-face interview by clinicians with experience in RLS diagnosis (MH, SH, HB) and was confirmed by the RLS Diagnostic Index [17]. Patients completed the study questionnaires (see below) and were evaluated according to age, gender, medication, former and current treatment of RLS, satisfaction with the actual treatment, and comorbid disorders as noted in the medical history. Those patients that were not able to fill in the questionnaires (cognitively disabled or illiterates) were excluded from the study. Also patients with incompletely filled in questionnaires were not included in the centres Bremen and Schwerin. In the centre Freiburg, the questionnaires were inspected regarding missing data during the clinical investigation and were completed together with the patient if necessary. For the subgroup analyses, patients were classified according to their treatment status. The group of untreated patients comprised a) treatment naïve and b) currently untreated patients with treatment experience. The group of treated patients were a) patients who were satisfied with the actual treatment and b) patients who were dissatisfied with the actual treatment regimen. The group of dissatisfied patients was then assigned in each centre to three subgroups i) augmentation, ii) loss of efficacy, and iii) other side effects according to the judgement of the local investigator. At the time of data collection, diagnostic criteria and severity rating scales for augmentation were not established; therefore, augmentation severity was not evaluated in the study. The study was approved by the local ethics committee and all patients gave written informed consent.

### Questionnaires

The Symptom-Checklist-90-R (SCL-90-R [14,15]) is a validated 90-item multidimensional self-rating questionnaire originally developed to assess the psychopathology of psychiatric and medical outpatients and further extended to measure psychological distress in a wide range of populations. It assesses a broad range of physical and psychological symptoms that might have bothered or distressed the subjects in the past seven days. Each of the 90 items is rated on a 5-point scale (ranging from 0 to 4), with higher values indicating greater impairment. The items build nine sub-scales: somatisation, compulsivity, interpersonal sensitivity, depression, anxiety, hostility, phobic anxiety, paranoid ideation, and psychoticism. The Global Severity Index (GSI) is derived from all items and indicates the degree of overall psychological distress/impairment. Raw scores for the sub-scales and the GSI are calculated ranging between 0 - 4 (0 = no distress to 4 = maximal distress). These can be transformed into age and gender-specific normative values (T-value, normal range 50 ± 10, higher values indicating greater psychological distress) by using the standardisation reference table [14,15]. The sub-scales show satisfactory reliability in chronic pain patients who are similarly impaired as RLS patients. Cronbach's alpha range from α = 0.71 to α = 0.89, the GSI is very consistent with a Cronbach's alpha of α = 0.97 [18].

The Beck Depression Inventory-II (BDI-II [19]) is a 21 item self-rating scale for assessing the experience of depressive symptoms in the preceding seven days. The item-response scales range from 0 to 3, with higher scores indicating more severe depressive symptoms. The sum score can range from 0 to 63 points. A score ≥ 18 points indicates clinically relevant depression. Good to very good reliability (0.84 ≤ α ≤ 0.92) was reported for the BDI-II in psychiatric, chronic pain and non-clinical populations [20-23].

RLS severity was additionally assessed using the validated International RLS Severity Scale (IRLS; Cronbach's α = 0.93-0.95 [24]). The self-rating questionnaire includes ten items (responses ranging from 0 to 4) evaluating the symptom severity and the impact of symptoms on everyday life activities. A total score of 1 to 10 points indicates mild, 11 to 20 moderate, 21 to 30 severe, and 31 to 40 very severe RLS symptoms.

### Statistical analysis

Demographic characteristics were analysed using analysis of variance, Kruskal-Wallis test, and chi^2 ^test. The questionnaires were analysed with Mann-Whitney U tests in order to detect differences between patient groups. Sub-scales of the SCL-90-R of untreated patients were also compared with the reference scores of a German representative sample [25] using one sample t-tests. Spearman rank correlation was used for correlation analysis. No adjustment for multiplicity of statistical analyses was performed in this exploratory study.

## Results

### Patient population

Characteristics of the study population are shown in Table 1. Patients were 59.6 ± 12.9 years old, 65.7% were female. The mean IRLS score was 27.2 ± 7.7 and the BDI-II score was 13.0 ± 9.1. Age (p = 0.23), gender (p = 0.75), psychological symptoms as assessed by the BDI-II (p = 0.35), and RLS severity as assessed by the IRLS (p = 0.75) were not different in the three study centres.

**Table 1:** Psychometric data of the study population

	Untreated patients (N = 69)	Treated patients			
		
		Satisfied with the actual treatment (N = 35)	Dissatisfied with the actual treatment		
			
			Augmentation (N = 19)	Loss of efficacy (N = 35)	Side effects (N = 8)
**IRLS**	24.0 (8.8)	26.9 (5.6)	31.4 (4.1)**	31.6 (5.9)***	27.1 (5.8)

**BDI-II**	11.3 (8.9)	12.0 (8.4)	15.8 (8.3)*	16.1 (10.3)*	11.9 (5.4)

**SCL-90-R**					

GSI	0.7 (0.6)	0.7 (0.5)	1.0 (0.6)*	0.9 (0.6)	0.6 (0.3)

Somatisation	0.9 (0.6)	0.9 (0.6)	1.2 (0.6)*	1.2 (1.1)	0.8 (0.4)

Compulsivity	0.8 (0.7)	0.8 (0.8)	1.2 (0.8)*	1.2 (0.9)*	0.9 (0.4)

Insecurity in social contact	0.8 (2.7)	0.6 (0.6)	0.9 (0.6)*	06. (0.6)	0.5 (0.5)

Depression	0.7 (0.8)	0.8 (0.8)	1.0 (0.8)	1.0 (0.8)*	0.8 (0.4)

Anxiety	0.6 (0.1)	0.7 (0.5)	0.9 (0.6)*	0.8 (0.6)	0.4 (0.3)

Hostility	0.4 (0.5)	0.5 (0.5)	0.8 (0.6)**	0.6 (0.7)	0.4 (0.3)

Phobic anxiety	0.4 (0.6)	0.2 (0.4)	0.5 (0.7)	0.5 (0.6)*	0.2 (0.2)

Paranoid ideation	0.4 (0.5)	0.4 (0.5)	0.8 (0.7)**	0.6 (0.6)	0.3 (0.2)

Psychoticism	0.4 (0.7)	0.3 (0.7)	0.6 (1.0)	0.4 (0.5)	0.2 (0.2)

*: p < 0.05; **: p < 0.01; ***: p < 0.001

Raw scores of sub-scales and Global Severity Index (GSI) of the SCL-90-R are presented. Values are mean (SD). Statistically significant results refer to comparisons of each group with untreated patients.

The actual medication was levodopa in 46% of patients, dopamine agonists in 22%, a further 10% received combinations of two dopamine agonists, and 12% received dopaminergic substances that were combined with other treatments. Four percent of patients received opioids, 2% anticonvulsants, and 3% other unspecific treatments. Notably, in the augmentation group, all patients received dopaminergic treatment: levodopa (n = 8), dopamine agonists (n = 7), combination of both (n = 3), or a dopamine agonist with opioids (n = 1). Comorbid disorders were documented in 74% of patients: 63% in untreated and 81% in treated patients.

Iron deficiency was documented in two patients (one untreated patient and one patient with augmentation). It is noteworthy that at the time of data collection we did not routinely screen patients for iron deficiency. One patient had renal failure and was satisfied with her RLS treatment.

### Psychological characteristics of patients

SCL-90-R data are presented in Table 1. RLS patients revealed normative SCL-90-R scores in the upper normal range (T-values < 60; Table 2). Compared to a German representative population sample [25] we found in untreated patients elevated raw scores on the sub-scales somatisation (p < 0.001), compulsivity (p = 0.003), depression (p = 0.02), anxiety (p = 0.004), and on the Global Severity Index (GSI; p = 0.002). Normative values of these sub-scales were in the normal range indicating no clinically relevant abnormality.

**Table 2:** Normative values of the SCL-90-R sub-scales and GSI in the study population

	Untreated patients (N = 69)	Treated patients			
		
		Satisfied with the actual treatment (N = 35)	Dissatisfied with the actual treatment		
			
			Augmentation (N = 19)	Loss of efficacy (N = 35)	Side effects (N = 8)
**SCL-90-R**					

GSI	56.1 (10.2)	56.4 (11.0)	**62.2 (10.3)**	**61.0 (11.6)**	56.6 (4.0)

Somatisation	56.0 (9.9)	56.2 (9.5)	**61.8 (10.5)**	58.3 (12.6)	56.2 (4.6)

Compulsivity	56.1 (11.5)	56.0 (11.0)	**62.0 (11.7)**	**62.4 (12.2)**	59.6 (4.9)

Insecurity in social contact	53.0 (10.1)	55.0 (10.6)	59.9 (10.6)	56.4 (11.7)	53.3 (10.4)

Depression	54.1 (10.0)	56.5 (12.4)	59.4 (12.7)	**60.2 (12.5)**	58.4 (5.7)

Anxiety	56.0 (10.6)	57.0 (10.0)	**62.4 (8.3)**	**60.4 (9.5)**	54.4 (5.7)

Hostility	53.0 (9.7)	55.5 (9.3)	**61.0 (8.8)**	57.8 (11.7)	52.9 (8.6)

Phobic anxiety	51.8 (10.6)	48.5 (9.4)	56.6 (11.4)	56.1 (10.9)	50.1 (7.7)

Paranoid ideation	50.0 (9.4)	49.9 (9.3)	58.1 (9.5)	53.4 (10.6)	50.0 (6.2)

Psychoticism	53.7 (9.2)	52.3 (9.7)	57.8 (12.7)	55.9 (10.4)	53.4 (6.3)

Values are mean (SD). Bold letters indicate values above the normal range (T-values > 60), higher values indicate higher distress.

Considering the whole study population, the extent of psychological problems correlated with RLS severity (GSI of SCL-90-R and IRLS; r = 0.4; p < 0.001).

### Subgroup analyses

**Figure 1:**
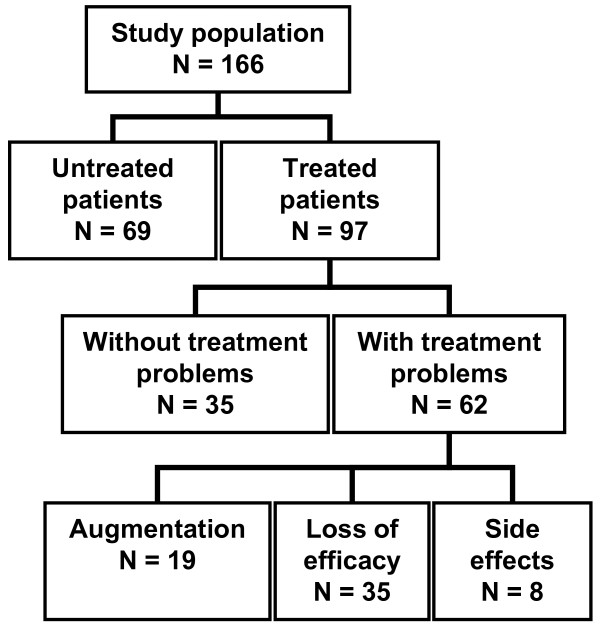
**Flow diagram of study population**.

A flow diagram of the study population is provided in Figure 1. Untreated patients were slightly younger than treated patients without treatment problems and treated patients with treatment problems (56.1 ± 12.9, 61.9 ± 9.6, 62.2 ± 13.6, respectively; p = 0.03), the gender distribution was comparable in the subgroups (p = 0.1).

**Figure 2:**
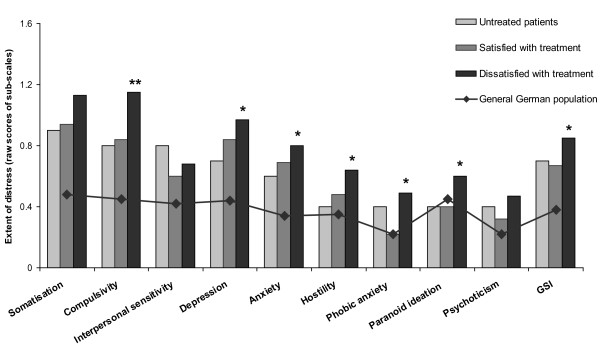
**SCL-90-R sub-scales and Global Severity Index in RLS patients**. Presented are the three patient groups (shaded bars): untreated patients, patients satisfied with the treatment and patients dissatisfied with their actual treatment. Patient groups with significantly higher scores compared with those of the group untreated patients are indicated with asterisks. SCL-90-R scores of a representative German population sample [25] are presented also (horizontal line). GSI: Global Severity Index. *: p < 0.05, **: p < 0.01.

SCL-90-R scores of treatment-naïve and at the time untreated patients but with treatment experience were comparable (0.16 ≤ p ≤ 0.83). The groups of treatment-naïve and at the time untreated patients were merged for the statistical analysis and are further reported as the group of untreated patients. Compared with these untreated patients, the treated group showed higher scores on the SCL-90-R sub-scales compulsivity (1.0 ± 0.8 vs. 0.8 ± 0.7, p = 0.044; raw data values), depression (0.9 ± 0.8 vs. 0.7 ± 0.9, p = 0.028), anxiety (0.8 ± 0.5 vs. 0.6 ± 0.6, p = 0.048), and hostility (0.6 ± 0.6 vs. 0.4 ± 0.5, p = 0.032) as well as on the IRLS (29.5 ± 5.9 vs. 24.0 ± 8.8, p < 0.001) and the BDI-II (14.2 ± 9.0 vs. 11.3 ± 8.9, p = 0.010). When analyzing the subgroups of treated patients, those dissatisfied with their treatment accounted for the higher IRLS scores (Table 1) and revealed the highest psychological distress. Compared with untreated patients, the SCL-90-R sub-scales compulsivity, depression, anxiety, hostility, phobic anxiety, paranoid ideation, and the GSI were elevated in these patients (Figure 2). The highest scores were seen in the sub-scales somatisation, compulsivity, depression, and anxiety. In this subgroup, augmented patients were those most affected by psychological symptoms compared with untreated patients (Table 1). Normative SCL-90-R scores of patients with augmentation and those with loss of efficacy were markedly elevated in the sub-scales compulsivity and anxiety (T-values > 60; Table 2), these patients were also those most severely affected by the RLS symptoms (IRLS: 31.4 ± 4.1 and 31.6 ± 5.9, Table 1).

Clinically relevant depression (BDI-II score ≥ 18) was present in 23% of the whole patient population. The largest proportion of patients with clinically relevant depressive symptoms was dissatisfied with treatment (29%). Depressive symptoms were most elevated in patients with augmentation or loss of efficacy (Table 1).

## Discussion

We investigated psychological distress in patients with RLS in a cross-sectional study. This study has two major findings: Firstly, RLS patients who are untreated show slightly elevated psychological distress in the domains somatisation, compulsivity, depression, and anxiety compared to representative values. Second, the psychological distress increases with the experience of frustrane treatments such as loss of efficacy and augmentation and can lead to clinically relevant psychological problems particularly in the domains of compulsivity and anxiety. The study yielded new evidence on psychological impairment of patients with RLS as to our knowledge no other study investigated the whole spectrum pf psychopathology in RLS. Of particular interest is our finding of elevated somatisation, which is frequently found in chronic disorders [18,26-30]. Corresponding to this finding, a recent study described a high rate of somatoform disorders (41%) and of chronic pain (34%) in RLS patients [31], and these comorbidities contributed to an unfavourable RLS treatment outcome [31]. A further interesting finding is the relatively high score for compulsive behaviour, particularly in treated patients. This finding is in line with recent observations reported in connection with the occurrence of impulse control disorders, such as pathological gambling, shopping addiction, and drug hoarding during dopaminergic treatment in Parkinson's disease [32] and RLS [33,34]. Reported drug hoarding and increased medication consumption that was associated with augmentation [34] corresponds to our observation of elevated compulsivity in augmented patients. Elevated depression and anxiety scores have been reported in RLS (for review see [35]), our findings are in line with these studies.

The psychological burden appears to be the highest in patients with augmentation followed closely by those experiencing loss of treatment efficacy. An explanation for this, though not specific to RLS, may be that frustration encountered during the course of treatment may promote feelings of helplessness and negative cognitions such as catastrophic thoughts.

The main limitation of the study is its cross-sectional design. Therefore, it remains difficult to judge whether poor long-term responders to treatment may be predisposed by psychological factors to the development of psychological problems or whether the treatment itself, including dopaminergic therapy, may impact psychological functioning. Longitudinal studies observing the change in burden experienced over time in routine care are needed. In future studies the influence of comorbid chronic disorders and intake of non-RLS specific medications should be considered. A more detailed assessment of treatment problems is also required. A selection bias may exist in the centres Bremen and Schwerin, where patients with incomplete questionnaires were not included in the study. Comparison of the populations in the centres revealed, however, no differences in the main characteristics such as age, gender, psychological symptoms, or RLS severity.

Severely affected RLS patients show psychological impairment with abnormalities in multiple psychological domains. These particularly interesting abnormalities should be considered in the treatment of RLS patients. For some severely affected patients, psychological support may be necessary. Patients can benefit from being educated in coping strategies that enable the patients to deal better with the disorder and prevent exacerbation of psychological symptoms [36,37]. Cognitive interventions may help in better coping with depressive and anxiety symptoms and mindfulness-based exercises [36,37] may reduce the sympathetic hyperactivity described in RLS [38]. In a pilot study, such strategies were applied successfully to a group of patients with mild to moderate RLS [36,37].

## Conclusions

In conclusion, patients with RLS show elevated psychological distress in multiple psychological domains. More severe RLS symptoms are associated with elevated psychological impairment. The psychological distress may contribute to an unfavourable treatment outcome and has to be taken into account in the treatment regimen of severely affected patients.

## Competing interests

The authors declare that they have no competing interests.

## Authors' contributions

MH, HB, SH, and RK conceived the study. MH, HB, and SH collected data. Statistical analysis was performed by RK and HS. MH and HS wrote the manuscript. MH, HB, SH, RK, and JB provided critical review. All authors read and approved the final manuscript.
